# Hepatoprotective Action of *Radix Paeoniae Rubra* Aqueous Extract against CCl_4_-Induced Hepatic Damage

**DOI:** 10.3390/molecules16108684

**Published:** 2011-10-17

**Authors:** Ruidong Li, Wenyuan Guo, Zhiren Fu, Guoshan Ding, You Zou, Zhengxin Wang

**Affiliations:** Department of Organ Transplantation, Changzheng Hospital, The Second Military Medical University, Shanghai 200003, China; Email: rdliczsh@yahoo.com.cn (R.L.); wyguoczsh@yahoo.com.cn (W.G.); zrfuczsh@yahoo.com.cn (Z.F.); gsdingczsh@yahoo.com.cn (G.D.); yzczsh@yahoo.com.cn (Y.Z.)

**Keywords:** hepatoprotection, rat, carbon tetrachloride, *Radix Paeoniae Rubra*, antioxidant

## Abstract

In the present study the capacity of *Radix Paeoniae Rubra* aqueous extract (RPRAE) as an antioxidant to protect against carbon tetrachloride (CCl_4_)-induced oxidative stress and hepatotoxicity in Wistar rats was investigated. Six groups of rats were used. *Radix Paeoniae Rubra aqueous extract* (100 or 200 or 300 mg/kg of bw) or bifendate (100 mg/kg of bw) were given daily by gavage to the animals on 28 consecutive days to elucidate the protective effects against CCl_4_-induced hepatotoxicity. The 20% CCl_4_/olive oil was gavage of gastric tube twice a week (on the third and seventh days of each week). The animals of normal control group were given only vehicle. The animals of CCl_4_-treated group were administered with CCl_4_ twice a week (on the third and seventh days of each week) and with vehicle on rest of the days. The test materials were found effective as hepatoprotective agents, as evidenced by plasma and liver biochemical parameters. Therefore, the results of this study show that *Radix Paeoniae Rubra aqueous extract* can protect the liver against CCl_4_-induced oxidative damage in rats, and the hepatoprotective effects might be correlated with its antioxidant and free radical scavenger effects.

## 1. Introduction

Oxidative stress contributes a decisive generating factor in the pathogenesis of acute and chronic liver diseases [1,2,3]. Carbon tetrachloride (CCl_4_) has been extensively used in animal models to explore chemical toxin-induced hepatic injury [4,5,6]. The metabolism of CCl_4_ catalyzed by liver microsomal cytochrome P450 rapidly overproduces free radicals that deplete hepatic glutathione and initiate a chain lipid peroxidation of the hepatocyte membrane. This ultimately results in the overproduction of reactive oxygen species (ROS) and hepatocyte injuries [7,8,9].

*Radix Paeoniae Rubra* (“Chi-shao” in Chinese) is one of the most widely used Chinese herbal drugs. *Radix Paeoniae Rubra* (RPR) has been introduced into the treatment of neural insult, after extensive pharmacological research and clinical trials [10]. The active compounds in RPR include paeoniflorin, benzoylpaeoniflorin, albiflorin, lactiflorin, oxypaeoniflorin, paeonin, paeoniflorigenone, paeonoside, paeonolide, paeonol, galloylpaeoniflorin, gallotannin. Natural remedies from medicinal plants are considered to be effective and safe alternative treatments for hepatotoxicity. Some previous studies have confirmed that RPR can prevent D-galactosamine induced mouse liver injury [11], BCG and LPS induced mouse immunological liver injury [12], CCl_4_-induced hepatic fibrosis [13,14], promote postoperative hepatocyte reproduction [15]. In this study, we tested the hepatoprotective action of *Radix Paeoniae Rubra aqueous extract* against the CCl_4_-induced hepatic damage.

## 2. Results

The effects of aqueous extract of *Radix Paeoniae Rubra* at three dose levels (100, 200 and 300 mg/kg, b.w.) on serum marker enzymes are shown in Table 1. Hepatic injury induced by CCl_4_ caused significant rise in marker enzymes AST, ALT, ALP activities and decrease in serum TP, Alb, G levels (*P* < 0.01). Administration of aqueous extract of *Radix Paeoniae Rubra* at three different dose levels attenuated the increased levels of the serum enzymes, produced by CCl_4_, and caused a subsequent recovery towards normalization almost like that of bifendate treatment.

**Table 1:** Effect of aqueous extract of *Radix Paeoniae Rubra* on serum AST, ALT and ALP activities.

Group	AST (U/L)	ALT (U/L)	ALP (U/L)
NC	193.21 ± 12.65	39.75 ± 2.09	152.77 ± 10.65
CCl_4_-treatment	401.43 ± 29.85 ^b^	307.47 ± 19.65 ^b^	518.36 ± 36.27 ^b^
CCl_4_+RPRAE (100 mg/kg b.w.)	314.06 ± 22.14 ^d^	263.16 ± 13.61 ^c^	403.75 ± 33.32 ^d^
CCl_4_+RPRAE (200 mg/kg b.w.)	251.63 ± 15.07 ^d^	194.09 ± 13.69 ^d^	284.18 ± 14.09 ^d^
CCl_4_+RPRAE (300 mg/kg b.w.)	192.86 ± 12.54 ^d^	74.03 ± 4.53 ^d^	170.39 ± 12.57 ^d^
CCl_4_+Bifendate (100 mg/kg b.w.)	217.53 ± 15.06 ^d^	121.47 ± 8.64 ^d^	206.41 ± 18.54 ^d^

^b^ *P* < 0.01, compared with NC group; ^c^* P* < 0.05, ^d^* P* < 0.01, compared with CCl_4_-treated group. Note: NC (normal control).

The hepatic cells in the CCl_4_-inducted group were found to suffer obvious fatty degeneration and necrosis and vacuole formation after CCl_4_ treatment. Administration of aqueous extract of *Radix Paeoniae Rubra* at doses of 100, 200 and 300 mg/kg for 28 days could reduce the fatty degeneration and necrosis hepatic injury score. This finding was consistent with the levels of the enzymes markers.

**Table 2:** Effect of aqueous extract of *Radix Paeoniae Rubra* on serum TP, Alb and G levels.

Group	TP (mg/L)	Alb (mg/L)	G (mg/L)
NC	88.32 ± 5.06	44.16 ± 2.08	35.16 ± 1.43
CCl_4_-treatment	42.07 ± 2.75 ^b^	30.15 ± 1.66 ^b^	13.65 ± 1.09 ^b^
CCl_4_+RPRAE (100 mg/kg b.w.)	69.35 ± 4.53 ^d^	37.12 ± 1.53 ^d^	22.41 ± 1.35 ^d^
CCl_4_+RPRAE (200 mg/kg b.w.)	74.62 ± 5.13 ^d^	43.62 ± 2.47 ^d^	28.94 ± 1.84 ^d^
CCl_4_+RPRAE (300 mg/kg b.w.)	86.01 ± 4.76 ^d^	50.49 ± 3.09 ^d^	34.27 ± 2.53 ^d^
CCl_4_+Bifendate (100 mg/kg b.w.)	71.54 ± 5.32 ^d^	47.53 ± 3.11 ^d^	32.61 ± 2.05 ^d^

^b^ *P* < 0.01, compared with NC group; ^d^* P* < 0.01, compared with CCl_4_-treated group.

Note: NC (normal control).

Liver index and hydroxyproline level in CCl_4_ treated rats were significantly higher than in control (liver index: 4.73 ± 0.24 *vs.* 3.15 ± 0.12; hydroxyproline: 481.63 ± 23.11 *vs.* 108.54 ± 8.54) as shown in Table 3. Liver index and hydroxyproline level in rats treated with aqueous extract of *Radix Paeoniae Rubra* and bifendate were significantly decreased. The effect was dose-dependent.

**Table 3:** Effect of aqueous extract of *Radix Paeoniae Rubra* on liver index and hydroxyproline levels.

Group	Liver index	Hydroxyproline (μg·g^−1^)
NC	3.15 ± 0.12	108.54 ± 8.54
CCl_4_-treatment	4.73 ± 0.24 ^b^	481.63 ± 23.11 ^b^
CCl_4_+RPRAE (100 mg/kg b.w.)	4.31 ± 0.25 ^c^	361.57 ± 23.07 ^d^
CCl_4_+RPRAE (200 mg/kg b.w.)	3.91 ± 0.19 ^d^	239.11 ± 16.74 ^d^
CCl_4_+RPRAE (300 mg/kg b.w.)	3.43 ± 0.12 ^d^	156.37 ± 12.14 ^d^
CCl_4_+Bifendate (100 mg/kg b.w.)	3.57 ± 0.19 ^d^	194.08 ± 18.62 ^d^

^b^ *P* < 0.01, compared with NC group; ^c^* P* < 0.05, ^d^* P* < 0.01, compared with CCl_4_-treated group.

Note: NC (normal control).

MDA level is widely used as a marker of free radical mediated lipid peroxidation injury. We measured MDA levels in the livers and the results are shown in Table 4. MDA levels in the CCl_4_-treated group were significantly higher than that in the normal control group (*P* < 0.01). Consistent with the serum levels of ALT, AST, and ALP, administration with aqueous extract of *Radix Paeoniae Rubra* significantly decreased CCl_4_-induced hepatic lipid peroxidation. The percentages of MDA levels in three doses of RPRAE-treated group (100, 200, 300 mg/kg) was significantly lower than that in the CCl_4_-treated group (*P* < 0.01). Bifendate also inhibited the elevating MDA levels upon CCl_4_ administration (Table 4). In addition, treatment with CCl_4_ also significantly decreased the GSH levels in the liver as compared to the normal control group. In contrast with CCl_4_-treated group, mice treated with aqueous extract of *Radix Paeoniae Rubra* (100, 200, 300 mg/kg) showed a significantly increase of GSH levels. These findings indicated that the free radicals being released in the liver were effectively scavenged by aqueous extract of *Radix Paeoniae Rubra*.

CCl_4_ treatment also resulted in the depletion of the hepatic antioxidant enzymes. The activities of SOD, CAT, GSH-Px and TAOC were depleted to 127.39 ± 10.54, 32.08 ± 0.14, 36.19 ± 0.22 and 2.13 ± 0.12 respectively of the control (Table 4). The treatment of rats with aqueous extract of *Radix Paeoniae Rubra* provided protection against the depletion in activities of these enzymes (Table 4). This shows aqueous extract of *Radix Paeoniae Rubra* to protect rats against oxidative injury by maintaining the levels of these enzymes even after CCl_4_ treatment. Likewise, bifendate treatment also enhanced liver antioxidant enzymes activities in CCl_4_-trated rats. CCl_4_ induced inhibition in SOD, CAT, GSH-Px and TAOC may probably be due to their inactivation during the catalytic cycle. Under these conditions, aqueous extract of *Radix Paeoniae Rubra*, possessing potent free radical scavenging activity, may ameliorate the levels of H_2_O_2_ and O_2_^−^, consequently restoring enzyme activity. The components of aqueous extract of *Radix Paeoniae Rubra* may also induce the de novo synthesis of the antioxidant enzymes.

**Table 4:** Effect of aqueous extract of *Radix Paeoniae Rubra* on MDA and GSH levels and SOD, CAT, GSH-Px, TAOC activities.

Group	TBARS (nmol MDA/mg Prot)	GSH (mg/g Prot)	SOD (U/mg prot)	CAT (U/mg prot)	GSH-Px (U/mg prot)	TAOC (U/mg prot)
NC	234.42 ± 0.21	23.24 ± 1.33	363.14 ± 24.76	65.32 ± 0.43	52.11 ± 0.34	4.74 ± 0.32
CCl_4_-treatment	8.93 ± 0.65 ^b^	10.26 ± 1.07 ^b^	127.39 ± 10.54 ^b^	32.08 ± 0.14 ^b^	36.19 ± 0.22 ^b^	2.13 ± 0.12 ^b^
CCl_4_+RPRAE (100 mg/kg b.w.)	6.85 ± 0.35 ^d^	16.53 ± 1.25 ^d^	184.64 ± 13.29 ^d^	44.08 ± 0.25 ^d^	42.13 ± 0.27 ^c^	2.89 ± 0.19 ^c^
CCl_4_+RPRAE (200 mg/kg b.w.)	5.42 ± 0.24 ^d^	19.62 ± 1.66 ^d^	295.1 ± 21.64 ^d^	53.17 ± 0.31 ^d^	46.27 ± 0.21 ^d^	3.74 ± 0.14 ^d^
CCl_4_+RPRAE (300 mg/kg b.w.)	4.63 ± 0.3 ^d^	25.13 ± 1.72 ^d^	342.72 ± 22.54 ^d^	62.07 ± 0.36 ^d^	54.37 ± 0.33 ^d^	4.83 ± 0.31 ^d^
CCl_4_+Bifendate (100 mg/kg b.w.)	4.98 ± 0.37 ^d^	26.09 ± 2.01 ^d^	315.54 ± 25.01 ^d^	57.53 ± 0.4 ^d^	50.39 ± 0.38 ^d^	5.35 ± 0.29 ^d^

^b^ P < 0.01, compared with NC group; ^c^ P < 0.05, ^d^ P < 0.01, compared with CCl_4_-treated group.

Note: NC (normal control).

## 3. Discussion

Since the changes associated with CCl_4_-induced liver damage are similar to those of acute viral hepatitis [16], CCl_4_-mediated hepatotoxicity was chosen as the experimental model. The ability of a hepatoprotective drug to reduce the injurious effects or to preserve the normal hepatic physiological mechanisms which that have been disturbed by a hepatotoxin, is the index of its protective effects [17]. Liver damage induced by carbon tetrachloride (CCl_4_) involves biotransformation of free radical derivatives, increased lipid peroxidation and excessive cell death in liver tissue [18]. The toxic effects of CCl_4_ on liver have been extensively studied [19,20,21,22]. Serum AST, ALT are the most sensitive biomarkers used in the diagnosis of liver diseases [23]. During hepatocellular damage, varieties of enzymes normally located in the cytosol are released into the blood flow. Their quantification in plasma is useful biomarkers of the extent and type of hepatocellular damage [24]. Hepatocellular necrosis leads to elevation of the serum marker enzymes, which are released from the liver into blood [25]. Increased levels of ALT, AST, ALP, TP, Alb and G are conventional indicators of liver injury [26].

For the therapeutic strategies of liver injury and disease, it is important to find antioxidant compounds that are able to block liver injuries through free radicals generated due to toxic chemicals. Therefore, the present study speculated that the aqueous extract of *Radix Paeoniae Rubra* protects against diseases that are caused by reactive oxygen species (ROS) because it has radical scavenging ability based on its antioxidant activity against CCl_4_ in rats. In this study, rats treated with CCl_4_ developed significant hepatic damage as manifested by a significant increase in activities of AST, ALT, ALP, and decrease in levels of TP, Alb and G that are indicators of hepatocyte damage and loss of functional integrity. These findings may reflect that 28 days were enough to detect abnormal AST, ALT, ALP, TP, Alb and G production in serum in this animal model. Centrilobular necrosis, lymphocytes infiltration and steatosis were also apparent in this study. Administration of the aqueous extract of *Radix Paeoniae Rubra* significantly reverse these abnormal indexs. This indicated that aqueous extract of *Radix Paeoniae Rubra* can ameliorate hepatic function of hepatic injury induced by CCl_4_. Wang *et al*. reported that the aqueous extract of *Radix Paeoniae Rubra* could decrease serum AST, ALT levels in mice treated by D-galactosamine [11]. These findings are in agreement with our previous results.

Liver hydroxyproline levels were measured as an index of hepatic collagen content. Hepatic stellate cells activated by CCl_4_-induced oxidative stress overproduce extracellular matrix components and tissue inhibitors of metalloproteinases (TIMPs). These TIMPs inhibit collagenases, which normally degrade collagen. Thus, the rise in collagen deposition occurs [27]. In our study, liver hydroxyproline level showed a massive increase after CCl_4_ long-term administration. Treatment with the aqueous extract of *Radix Paeoniae Rubra* and bifendate decreased its level significantly. Our finding supported other authors’ observations that long-term administration of CCl_4_ resulted in marked increase in the level of hydroxyproline [28,29]. Co-treatment with antioxidants or plant extracts [29,30] prevented the increase in hepatic hydroxyproline level significantly.

On the other hand, the metabolic transformation of CCl_4_ by the NADPH-cytochrome P450 system to form the trichloromethyl radical CCl_3_• and trichloromethyl peroxy radical CCl_3_OO•, may also play an important role in the process of lipid peroxide which has the major role in the degenerative processes of the tissues [31,32,33]. Enhanced lipid peroxidation may associated with depletion of antioxidant, GSH in the heart of CCl_4_ treated rats which is a characteristic observation in CCl_4_-intoxicated rats [34,35]. GSH is important in effecting detoxification of the reactive metabolites of cells; tissue necrosis is initiated when reserves of GSH are markedly depleted [34,36]. Thus, the reduced (relative to normal) levels of GSH observed in the heart tissues of rats (administered CCl_4_) might reflect to increased oxidative damage.

Antioxidant enzymes activities in liver of CCl_4_-treated group rats were significantly lower than those in normal control group. Antioxidant enzymes (SOD, GPx and catalase) represent one protection against oxidative tissue-damage [37]. The cleavage of CCl_4_ leads to the formation of highly unstable free radicals (CCl_3_• or CCl_3_O_2_•), to initiate peroxidation [38]. Thus, the inhibition of the generation of free radicals retards CCl_4_-induced lipid peroxidation. The administration of the aqueous extract caused an elevation of the levels of CAT, GSH-Px, TAOC and SOD and decreased the level of TBARS in liver, suggesting that it can restore these antioxidant enzymes and/or activate enzyme activity in the damage caused by CCl_4_.

## 4. Experimental

### 4.1. Material

Roots of *Radix Paeoniae Rubra* was purchased from a herb market in Shanghai city, China in 2010 and identified by Dr. FL Ma, Department of Plant Biology (Shanghai Jiaoatong University, China). A voucher specimen was deposited in the laboratory of the School of Pharmacology, under the number 20100541.

### 4.2. Preparation of Aqueous Extract of Roots of *Radix Paeoniae Rubra*

Roots of *Radix Paeoniae Rubra* were dried under shade and pulverized. *Radix Paeoniae Rubra* root powder (600 g) was suspended in distilled water (5,000 mL), heated and boiled under reflux for 120 min. The extracts were filtered through Whatman No. 2 filter paper, and the filtrate was pooled and concentrated using a rotary evaporator at 50 °C and freeze-dried at −70 °C. The yield of dried aqueous extract of roots of *Radix Paeoniae Rubra* (RPRAE) was 13.85% (w/w).

### 4.3. Treatment of Animals

Male Wister rats (200–220 g/rat) were used in the experiments. These rats were provided with laboratory rodent diet and water ad libitum and randomly divide into six groups (eight rats/group): normal control (NC), CCl_4_-treated group, three RPRAE-treated groups and a bifendate-treated group (positive control). To study the protective effect against the CCl_4_-induced chronic hepatic injury and oxidative stress, *Radix Paeoniae Rubra* aqueous extract (100 or 200 or 300 mg/kg of bw) or bifendate (100 mg/kg of bw) were given daily by gavage to the animals on 28 consecutive days to elucidate the protective effect against CCl_4_-induced hepatotoxicity. The 20% CCl_4_/olive oil was gavage of gastric tube twice a week (on the third and seventh days of each week). The animals of normal control group were given only vehicle. The animals of CCl_4_-treated group were administered with CCl_4_ twice a week (on the third and seventh days of each week) and with vehicle on rest of the days. In the 29th day, each rat was anesthetized with ethyl ether, then weighed, blood drawn and the organ tissues taken out. Blood samples were collected for the assays of aspartate aminotransferase (GST), alanine aminotransferase (GLT), alkaline phosphatase (ALP) activities and total protein (TP), albumin (Alb), globulin (G) levels. The livers were excised from the animals and assayed for the reduced glutathione (GSH) level, antioxidant enzymes activity, malondialdehyde (MDA) formation and pathological histology, according to the procedures described bellowed. The liver index should be calculated as followings: (liver wet weight/body weight) ×100. All experimental procedures involving animals were conducted in accordance with National Institutes of Health (NIH). This experiment was approved by the Institutional Animal Care and Use Committee of China.

### 4.4. Histopathological Examination

Hepatic tissues of rats were collected from the same lobe of the livers and fixed in 10% neutral formalin solution. Hepatic tissue was dehydrated and embedded in paraffin. Cross-sections were stained using haematoxylin and eosin (H & E) for photomicroscopic observation.

### 4.5. Biochemical Analysis

Conventional liver biochemical tests (AST, ALT, ALP) were performed on a clinical chemistry automatic analyzer ADVIA 1650 (Bayer Diagnostics, Tarrytown, NY, USA). AST, ALT, and ALP were measured using commercial AST, ALT and ALP kits (Bayer Diagnostics).

The serum total concentrations of total protein (TP), albumin (ALB), and globulin (G) were measured by using commercial kits (Labtest Diagnóstica, Minas Gerais, Brazil) on the Express Plus biochemical analyzer (Ciba-Corning Diagnostics, Medfield, Massachusetts, USA). Hydroxyproline content in the liver was monitored using a diagnostic kit (Jiancheng Bioengineering Institute, Nanjing, China).

Hepatic thiobarbituric acid reactive substances (TBARS) was assayed by the method of Ohkawa *et al.* [39] using the thiobarbituric acid reaction, except that 1.0 mM EDTA was added to the reaction medium. The concentration of hepatic TBARS is expressed as malondialdehyde (MDA) equivalents. Hepatic GSH was assayed by the method of Sedlak and Lindsay [40] using Ellman’s reagent. In this assay, GSH was used as a standard.

The catalase (CAT) activity was determined according to the Aebi method [41]. The rate of H_2_O_2_ decomposition was followed by monitoring absorption at 240 nm. One unit of CAT activity is defined as the amount of enzymes required to decompose 1 μmol of hydrogen peroxide in 1 min. The enzyme activity was expressed as μmol H_2_O_2_ consumed/min/mg protein. Superoxide dismutase (SOD) activity was estimated according to the method of Beauchamp and Fridovich [42]. The developed blue colour in the reaction was measured at 560 nm. Units of SOD activity were expressed as the amount of enzyme required to inhibit the reduction of NBT by 50% and the activity was expressed as U/mg protein. Glutathione peroxidase (GSH-Px) activity assayed using the method of Chiu *et al.* [43] in liver extracts. The level of total antioxidant capacity (TAOC) was measured by the method of ferric reducing/antioxidant power assay [44].

### 4.6. Statistical Analysis

The results of hepatoprotective effect were recorded for individuals within all groups. All data were presented as mean ± SD. Results were statistically analyzed by one-way ANOVA using SPSS10.0 statistical software. A value of *P* < 0.05 was considered significant.

## 5. Conclusions

Our results demonstrate a very good protective effect of *Radix Paeoniae Rubra* against CCl_4_-induced liver injury, which is probably due at least partly to its antioxidative properties, scavenging CCl_4_-associated free radicals. However, the mechanism of the hepatoprotective activity of *Radix Paeoniae Rubra* remains still to be proven. *Radix Paeoniae Rubra* might be useful as a natural ingredient for the prevention of oxidative damage in liver cells and tissues.
